# Multicomponent analysis of stripping voltammograms by target transformation factor analysis

**DOI:** 10.1155/S1463924696000193

**Published:** 1996

**Authors:** Ling Gao, Shouxin Ren

**Affiliations:** Department of Chemistry Inner Mongolian University Inner Mongolia Huhehot 010021 China


Journal of Automatic Chemistry, Vol. 18, No. 5 (September-October 1996), pp. 175-179

### Multicomponent analysis of stripping voltammograms by target transformation factor analysis

**Ling Gao and Shouxin Ren**

*Department of Chemistry, Inner Mongolian University, Huhehot 010021, Inner Mongolia, China*

*Target transformation factor analysis was studied for simultaneous determination of four simulated stripping voltammograms. A Gaussian function was used to generate synthetic stripping voltammograms. Two programs, SPGRELEP and ELECTTFA, were designed to perform voltammogram simulation and target transformation factor analysis. The method made use of full information of voltammogram and matrix data processing. The ELECTTFA program was used to determine the number of components, to identify the components and to yield a quantitative analysis of unknowns. Experimental results showed the method to be successful.*

### Introduction

Chemometric methods have been applied to improve results in the multicomponent analytical techniques in spectroscopy [1-3], but they have rarely been used in electrochemistry. Target transformation factor analysis (TTFA) is a technique that is especially valuable for achieving meaningful transformations of the abstract factors obtained by abstract factor analysis (AFA) [4]. TTFA can be used to yield both qualitative and quantitative information. It also can be used to decide whether or not a suspected substance is present in the mixture and to deduce the composition of each mixture. This method has been applied to spectroscopic, nuclear magnetic resonance, chromatography and kinetic analysis [5, 6]; attention has been paid, however, to applying TTFA to voltammetry. Many voltammetric techniques are of a local character, using only the peak currents or a limited number of points around the peak, thus losing much of the information contained in the remaining parts of the voltammogram. This paper describes the improvement of multicomponent determinations using the full information of voltammogram and matrix data processing.

### Theoretical background and program algorithms

### *Building the data matrix $D$ $(P, S)$*

The original data matrix, $D$, contains the anodic current response at $P$ voltage values of $S$ mixtures; each voltammogram being a column and the data taken at the same voltage being a row.

### *Abstract factor analysis of the experimental data matrix $D$*

The covariance matrix $Z(D^TD)$ can be subjected to singular value decomposition for calculation of the eigenvalues and eigenvectors, $Z = USV^T$, where $S$ is a diagonal matrix of the eigenvalues and $U$ is the matrix of eigenvectors. The $D$ matrix can be represented as the product of two matrices: $D = RC$, where $R(P, F)$ are the current profiles of the present species and $C(F, S)$ their concentration matrix (where $F$ is the number of components). It is easy to compute: $C = U^T$ and $R = DU$ due to the orthonormality of $U^{-1}$ and $U^T$. The number of principal components is estimated by several criteria, which are based on the theory of error in factor analysis [7]. Not all of the eigenvectors produced convey useful information; some are due to noise. After deleting the insignificant eigenvectors $R$ and $C$ become $R_1$ $(P, a)$ and $C_1$ $(a, S)$, where $a$ is the number of principal components.

### *Target transformation*

The abstract matrices must be rotated into significant real matrices. This oblique rotation is accomplished by transforming matrix $T$. The matrix $T$ can be determined by using the target vector $R_2$ from the equations $T = (R_1^TR_1)^{-1}R_1^TR_2$ or $T = S_1^{-1}R_1^TR_2$, and then the concentrations and the anodic current profiles of sample component are given by $C_0 = T^{-1}C_1$ and $R_0 = R_1T$, respectively. In traditional factor analysis, the current profiles of pure components are always used as the $R_2$ matrix to calculate $T$. This may cause errors due to interaction between the components. To overcome these, an $R_2$ matrix, calculated from the standard mixtures by classical least squares analysis $R_2 = D_3C_3^T(C_3C_3^T)^{-1}$, and a non-zero intercept added to each voltage value, are used as the target vector instead of the pure component standards. $C_3$ is the concentration matrix of the standard mixtures, $D_3$ is the response matrix of the standard mixtures.

### *Target testing*

The aim of target testing is to decide whether a proposed target can be accepted as a real factor. The criteria used for accepting/rejecting target vectors is based on the similarity between the target vector and the predicted target vector. Malinowski and Howery suggested using the *SPOIL* function to decide whether a proposed target is acceptable or not [4]. The SPOIL function is defined as the ratio of the real error in the target vector (*RET*) to the real error in the predicted vector (*REP*):

0142-0453/96 $12.00 © 1996 Taylor & Francis Ltd.

*SPOIL* = *RET*/*REP*. The apparent error in the test vector (*AET*) is calculated using:

$$AET = \left[\frac{\sum_{i=1}^{a} (R_0 - R_2)^2}{a}\right]^{1/2}.$$

*REP* is defined as $REP_i = RE(i)\,\|T_i\|$ with $i = 1, 2, \ldots, a$. Where $\|T_i\|$ is the Euclidean norm of the vector; *RET* is then evaluated by $RET = [(AET)^2 - (REP)^2]^{1/2}$. Malinowski and Howery divided the value of the SPOIL function into three regions: (1) an acceptable region (0·0–3·0); (2) a fair region (3·0–6·0); and (3) an unacceptable region (>6·0).

*Voltammogram simulation*

A Gaussian peak shape is assumed for the voltammograms of the pure component with peak position *M*, peak height *C*, and widths *W*:

$$\mathrm{cur}(i) = C(i)\exp\{-[(vol - M_1)/W_1]^2\}$$

The '*vol*' denotes the voltage values of the full voltammogram and the symbol *i* denotes the component number. The second part of the simulation consists of generating the measurement noise (*Nos*). The measurement noise is assumed to be normally distributed with zero mean:

$$E[Nos(vol_k)] = 0_{\text{for all Vol}_k}$$

and white with variance $R(\mathrm{Vol}_k)$:

$$E[Nos(vol_k)\cdot Nos(vol_k)] = R(vol_k)\delta_{k,j}$$

$$\delta_{k,j} = 1 \quad \text{if } k = j$$

$$\delta_{k,j} = 0 \quad \text{if } k \neq j$$

*E*[ ] denotes the expectation of the quantity within the brackets, $\delta$ is Kronecker's symbol.

Two programs, SPGRELEP and ELECTTFA, which are based on the algorithms were designed to perform voltammogram simulation and TTFA.

### Experimental

*Instrument*

A GW 286 EX/16 microcomputer with a maths coprocessor was used for the calculations.

*Simulated voltammograms*

Simulated data were used to evaluate the potential of TTFA for stripping voltammograms. The peak potentials of copper, lead, cadmium and zinc had the values of −0·35, −0·53, −0·70 and −1·10 V (VS.SCE). The peak widths of the four components were 0·05 V. Gaussian distributed noise was added to the voltammograms to simulate experimental noise as 1% of the average signal value. The background was assumed to be normally distributed with zero mean; the peak height of the background was 0·05 ppm. Taking background as baselines, the simulated voltammograms of nine unknown samples and baselines were plotted as shown in figure 1.

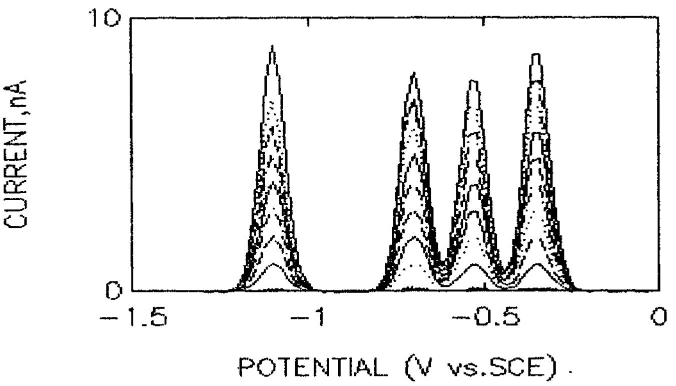

*Figure 1. Simulated voltammograms of the unknown samples.*

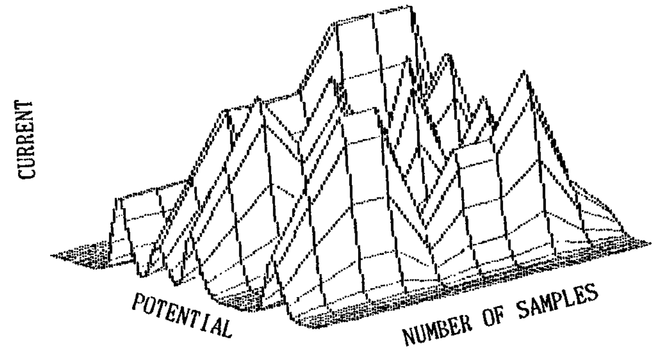

*Figure 2. Three-dimensional plot of the standard data matrix $D_3$.*

The voltammograms of the standard samples were simulated in the same way as the unknown samples. Figure 2 is a three-dimensional plot of the simulated standard data matrix $D_3$.

*Three-level orthogonal array design of standard samples*

For any orthogonal array design, a matrix, which consists of columns and rows, with various numbers at the intersections of each column and row, must be constructed. Table 1 displays an $L_9(3^4)$ matrix. This is a three-level orthogonal array matrix which is made up of four columns and nine rows. Each column represents a factor, which is an independent variable, and each row represents an experimental trial. The numbers at the intersections indicate the level settings that apply to

*Table 1. The selection of the concentrations of standard sample (ppm).*

| *No.* | 1 | 2 | 3 | 4 | Cu | Pb | Cd | Zn |
|---|---|---|---|---|---|---|---|---|
| 1 | 1 | 1 | 1 | 1 | 4 | 2 | 3 | 3 |
| 2 | 1 | 2 | 2 | 2 | 4 | 5 | 6 | 6 |
| 3 | 1 | 3 | 3 | 3 | 4 | 8 | 9 | 9 |
| 4 | 2 | 1 | 2 | 3 | 7 | 2 | 6 | 3 |
| 5 | 2 | 2 | 3 | 1 | 7 | 5 | 9 | 6 |
| 6 | 2 | 3 | 1 | 2 | 7 | 8 | 3 | 9 |
| 7 | 3 | 1 | 3 | 2 | 10 | 2 | 9 | 9 |
| 8 | 3 | 2 | 1 | 3 | 10 | 5 | 3 | 3 |
| 9 | 3 | 3 | 2 | 1 | 10 | 8 | 6 | 1 |

*Table 2. Results of the factor analysis on the unknown samples.*

| $N$ | EV | RE | IND | XE | IE | ER | REV | Frac |
|---|---|---|---|---|---|---|---|---|
| 1 | 3·1797E+3 | 2·9521E−1 | 4·6127E−3 | 2·7833E−1 | 9·8404E−2 | 1·1519E+2 | 5·3531E+0 | 9·8573E−1 |
| 2 | 2·7604E+1 | 1·9963E−1 | 4·0741E−3 | 1·7606E−1 | 9·4106E−2 | 2·5928E+0 | 5·3084E−2 | 8·5573E−3 |
| 3 | 1·0900E+1 | 1·4003E−1 | 3·8898E−3 | 1·1434E−1 | 8·0848E−2 | 1·3771E+0 | 2·3764E−2 | 3·3004E−3 |
| 4 | 7·7310E+0 | 1·0195E−2 | 4·0781E−4 | 7·5991E−3 | 6·7969E−3 | 7·0926E+2 | 2·0452E−2 | 2·3966E−3 |
| 5 | 1·0900E−2 | 9·4150E−3 | 5·8844E−4 | 6·2767E−3 | 7·0175E−3 | 1·2814E+0 | 3·5161E−5 | 3·3791E−6 |
| 6 | 8·5067E−3 | 8·6734E−3 | 9·6371E−4 | 5·0076E−3 | 7·0818E−3 | 1·4724E+0 | 3·4863E−5 | 2·6371E−6 |
| 7 | 5·7776E−3 | 8·3109E−3 | 2·0777E−3 | 3·9187E−3 | 7·3296E−3 | 1·0786E+0 | 3·2098E−5 | 1·7911E−6 |
| 8 | 5·3566E−3 | 7·5487E−3 | 7·5487E−3 | 2·5162E−3 | 7·1170E−3 | 1·4243E+0 | 4·5395E−5 | 1·6606E−6 |
| 9 | 3·7609E−3 | | | | | | 6·4843E−5 | 1·1659E−6 |

the factors for the experimental trials. From this matrix it can be noted that each of four columns is varied over three level setting, each level setting repeats three times, and thus a total of $3 \times 3 = 9$ experimental trials are necessary for each column. Furthermore, in any two columns, the horizontal combinations of any two level numbers appear at the same number of times. That is, each combination of the nine ordered pairs (1, 1), (1, 2), (1, 3), (2, 1), (2, 2), (2, 3), (3, 1), (3, 2), (3, 3) appears exactly once. The above features of the $L_9(3^4)$ matrix provide orthogonality among all the four columns. Three-level orthogonal array design with the $L_9(3^4)$ matrix provides a systematic procedure for selecting the concentration of the training set samples (see table 1).

### Results and discussion

### *Determination of the number of factors*

Eight criteria were used to calculate the number of factors and the results are shown in table 2. The IND function reached a minimum at $N = 4$; the magnitude of the first four eigenvalues were larger than those of 5–9; and a maximum of the eigenvalue ratio (ER) function appeared at 4. The magnitude of the reduced eigenvalues (REV) decreased rapidly until $N = 4$, then it stabilized. The minimum of the Frac function appeared at 5; after 5 the change in Frac was quite small (the appropriate number of eigenvectors is one less than that giving the minimum Frac value). Both the IE and XE functions show a sharp drop between the first and fourth eigenvectors and then they level off. A plot of logarithm of REV versus factor number is shown in figure 3. The REVs of factor 1–4 are clearly much larger than the rest. Some idea of the information contained in the various factors can be obtained by inspecting the corresponding eigenvector. The number of significant eigenvectors is determined by plotting them as a function of the voltage. From these figures, it is fairly obvious that the eigenvectors of the first four factors represent what appears to be linear combinations of the original pure component voltammograms. In contrast, the last five factors look like noise. Figures 4 and 5 show the eigenvector corresponding to factor 4 and 5, respectively. The fourth eigenvectors provide significant information, and the fifth look like noise. From these criteria, it was concluded that four significant components were present.

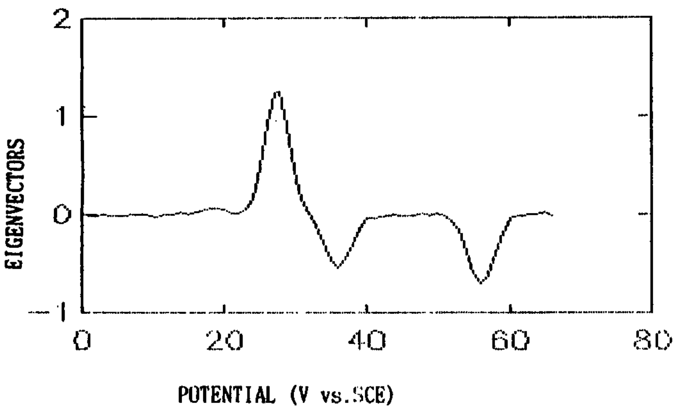

*Figure 4. Plot of the fourth eigenvectors of matrix D.*

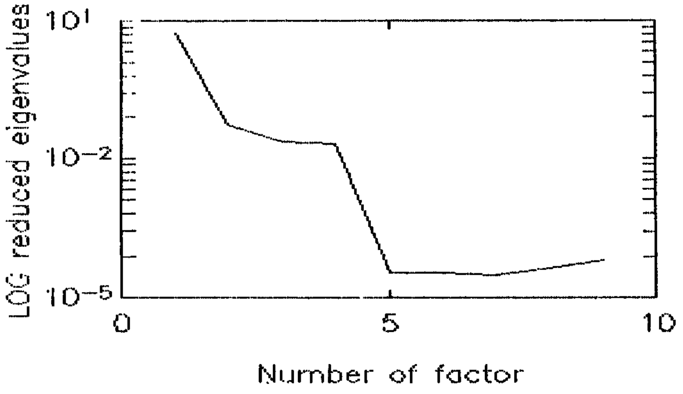

*Figure 3. Log REV versus factor number for matrix D.*

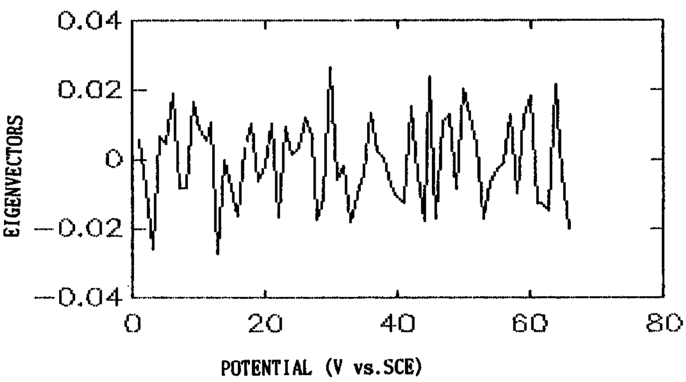

*Figure 5. Plot of the fifth eigenvectors of matrix D.*

*Table 3. Validation of target vector.*

| | Cu | Pb | Cd | Zn |
|---|---|---|---|---|
| AET | 0·0182 | 0·0227 | 0·0158 | 0·0158 |
| REP | 0·1319 | 0·1107 | 0·0484 | 0·0044 |
| RET | 0·1306 | 0·1083 | 0·0457 | 0·0152 |
| *SPOIL* | 0·9904 | 0·9788 | 0·9453 | 3·4496 |

### *Determination and validation of target vector*

According to the algorithms, the target matrix $R_2$ was calculated from the standard samples by the least squares method. The *SPOIL* function was developed, based on the similarity between the target vector and the predicted target vector, to the validity and usefulness of a predicted target vector. In order to judge whether the predicted target vectors were a successful target, the *SPOIL* values were calculated and the results are given in table 3. The *SPOIL* values of Cu, Pb and Cd were below 3·0 and the value of Zn was a little over 3·0, indicating that all the predicted target vectors can be considered to be true targets.

### *Simultaneous determination of unknown samples*

The abstract factor matrices, $R_1$ and $C_1$, were calculated from data matrix $D$. Because the target vectors for the four metals were known from the standard samples, target transforming matrix $T$ was calculated as shown in table 4. By target transformation the concentration and the current profile matrix, $C_0$ and $R_0$, were found. Using the ELECTTFA program, the concentrations of unknown samples and their recoveries, as well as relative deviation, are listed in tables 5 and 6. The experimental results proved that target transformation factor analysis provides satisfactory results for simultaneous determination of synthetic stripping voltammogram.

*Table 4. The target transforming matrix T.*

| | | | |
|---|---|---|---|
| 0·0166 | 0·0144 | 0·0155 | 0·0163 |
| 0·0902 | −0·0175 | −0·2694 | 0·1802 |
| −0·4364 | 0·1775 | 0·0216 | 0·2674 |
| 0·0283 | 0·5247 | −0·2147 | −0·2863 |

### Conclusion

A method has been described for simultaneous determination of four stripping voltammograms. It can be used to analyse the whole voltammogram rather than just picking out a few characteristic values. The analysis of simulated stripping voltammograms shows how the method can be used to identify the components and to yield a quantitative analysis of each mixture. The target matrix is calculated

*Table 5. The concentration of unknown samples by ELECTTFA.*

| | Concentration (ppm) | | | | | | | | |
|---|---|---|---|---|---|---|---|---|---|
| | Sample number | | | | | | | | |
| Species | (1) | (2) | (3) | (4) | (5) | (6) | (7) | (8) | (9) |
| Cu | 0·9942 | 2·0048 | 2·9930 | 4·0054 | 6·0038 | 4·9981 | 7·0050 | 7·9906 | 9·0058 |
| Pb | 0·9972 | 2·9989 | 2·0036 | 3·0017 | 4·0087 | 5·0014 | 7·0028 | 6·0014 | 8·0042 |
| Cd | 2·0096 | 2·9948 | 1·0019 | 4·0079 | 5·0003 | 6·9986 | 5·9987 | 6·9948 | 8·0032 |
| Zn | 1·0052 | 1·9976 | 4·0046 | 3·0049 | 3·9989 | 6·0006 | 7·0046 | 8·0005 | 8·9908 |

*Table 6. The recoveries and relative deviations of the unknown samples.*

| | Recovery (%) | | | | | | | | |
|---|---|---|---|---|---|---|---|---|---|
| | Sample number | | | | | | | | |
| Species | (1) | (2) | (3) | (4) | (5) | (6) | (7) | (8) | (9) |
| Cu | 99·4237 | 100·2421 | 99·7653 | 100·1356 | 100·0633 | 99·9611 | 100·0718 | 99·8827 | 100·0644 |
| Pb | 99·7187 | 99·9623 | 100·1796 | 100·0565 | 100·2182 | 100·0275 | 100·0395 | 100·0237 | 100·0531 |
| Cd | 100·4804 | 99·8265 | 100·1941 | 100·1975 | 100·0064 | 99·9795 | 99·9786 | 99·9264 | 100·0396 |
| Zn | 100·5233 | 99·8776 | 100·1143 | 100·1645 | 99·9736 | 100·0097 | 100·0650 | 100·0688 | 99·8976 |
| | Relative deviation | | | | | | | | |
| | Sample number | | | | | | | | |
| Species | (1) | (2) | (3) | (4) | (5) | (6) | (7) | (8) | (9) |
| Cu | −0·0058 | 0·0024 | −0·0023 | 0·0014 | 0·0006 | −0·0004 | 0·0007 | −0·0012 | 0·0006 |
| Pb | −0·0028 | −0·0004 | 0·0018 | 0·0006 | 0·0022 | 0·0003 | 0·0004 | 0·0002 | 0·0005 |
| Cd | 0·0048 | −0·0017 | 0·0019 | 0·0020 | 0·0001 | −0·0002 | −0·0002 | −0·0007 | 0·0004 |
| Zn | 0·0052 | −0·0012 | 0·0011 | 0·0016 | −0·0003 | 0·0001 | 0·0007 | 0·0007 | −0·0010 |

from the standard samples by least squares, instead of the pure component standard. The number of components is determined by inspection of both computed results and figures. The method was testified by simulated voltammograms with satisfactory results.

### Acknowledgement

The authors would like to thank the National Natural Science Foundation of China for financial support.

### References

1. Windig, W., *Chemometrics and Intelligent Laboratory system*, **23** (1994), 71.

2. Xie, Y., Liang, Y. and Yu, R., *Analytica Chimica Acta*, **272** (1993), 61.

3. Kubista, M., Sjoback, R., and Nygren, J., *Analytica Chimica Acta*, **302** (1995), 121.

4. Malinowski, E. R. and Howery, D. E., *Factor Analysis in Chemistry* (Wiley, New York, 1985).

5. Wirsz, D. F. and Blades, M. W., *Talanta*, **37** (1990), 39.

6. Cladera, A., Gomez, E., Estela, J., and Cerda, V., *Analytical Chemistry*, **65** (1993), 707.

7. Ren, S. and Gao, L., *Journal of Automatic Chemistry*, **17** (1995), 115.

